# Telomeres and Telomerase: Role in Marek’s Disease Virus Pathogenesis, Integration and Tumorigenesis

**DOI:** 10.3390/v9070173

**Published:** 2017-07-04

**Authors:** Ahmed Kheimar, Renato L. Previdelli, Darren J. Wight, Benedikt B. Kaufer

**Affiliations:** 1 Institut für Virologie, Freie Universität Berlin, Robert von Ostertag-Straße 7–13, 14163 Berlin, Germany; ahmed1985@zedat.fu-berlin.de (A.K.); previnato@zedat.fu-berlin.de (R.L.P.); d.wight@fu-berlin.de (D.J.W.); 2 Department of Poultry Diseases, Faculty of Veterinary Medicine, Sohag University, Sohag 82424, Egypt

**Keywords:** herpesvirus, Marek’s disease virus (MDV), telomeres, telomerase, integration, tumorigenesis

## Abstract

Telomeres protect the ends of vertebrate chromosomes from deterioration and consist of tandem nucleotide repeats (TTAGGG)_n_ that are associated with a number of proteins. Shortening of the telomeres occurs during genome replication, thereby limiting the replication potential of somatic cells. To counteract this shortening, vertebrates encode the telomerase complex that maintains telomere length in certain cell types via de novo addition of telomeric repeats. Several herpesviruses, including the highly oncogenic alphaherpesvirus Marek’s disease virus (MDV), harbor telomeric repeats (TMR) identical to the host telomere sequences at the ends of their linear genomes. These TMR facilitate the integration of the MDV genome into host telomeres during latency, allowing the virus to persist in the host for life. Integration into host telomeres is critical for disease and tumor induction by MDV, but also enables efficient reactivation of the integrated virus genome. In addition to the TMR, MDV also encodes a telomerase RNA subunit (vTR) that shares 88% sequence identity with the telomerase RNA in chicken (chTR). vTR is highly expressed during all stages of the virus lifecycle, enhances telomerase activity and plays an important role in MDV-induced tumor formation. This review will focus on the recent advances in understanding the role of viral TMR and vTR in MDV pathogenesis, integration and tumorigenesis.

## 1. Introduction

Marek’s disease virus (MDV) is a highly oncogenic alphaherpesvirus that infects chickens and causes vast economic losses in the poultry industry worldwide [1]. MDV, also known as gallid herpesvirus type 2 (GaHV-2), causes a variety of clinical symptoms including neurological disorders and immunosuppression, but is most renowned for its oncogenic potential [2]. The virus efficiently induces malignant T-cell lymphomas and is considered to be the most prevalent clinically-diagnosed cancer in animals [3]. MDV infection of susceptible chickens can result in a mortality rate of up to 100%. However, severity of disease and mortality are dependent on the genetic background of the host and the virulence of the virus strain [4]. A number of viral factors have been identified that contribute to pathogenesis and tumor formation including the major oncoprotein Meq (Marek’s EcoRI-Q-encoded protein) [5], the viral interleukin-8 (vIL-8) [6,7], MDV-encoded miRNAs [8,9] and numerous small putative open reading frames (ORFs) of unknown function [10,11]. Intriguingly, MDV and other herpesviruses harbor telomeric repeats (TMR) at the ends of their genomes [12,13]. In addition, MDV encodes a subunit of the telomerase complex, a viral telomerase RNA (vTR) [14,15,16]. In this review, we will discuss the role of viral TMR in the telomere integration of MDV and other herpesviruses, followed by the role of vTR in MDV pathogenesis and tumorigenesis.

## 2. Marek’s Disease Virus Integration

In infected chickens, MDV productively replicates in B-cells that transfer the virus to T-cells [1,17]. The virus subsequently establishes latency in CD4+ T-cells and integrates its genome into the telomeres of host chromosomes [18,19]. In addition, MDV is able to transform infected CD4+ T-cells, which ultimately leads to fatal lymphoma in visceral organs. Intriguingly, both latently infected and MDV-induced tumor cells harbor the integrated virus genome in the telomeres of multiple host chromosomes [20,21]. The integrated virus genome has been observed in up to 15 chromosomes in tumor cells [19], suggesting that the integration process is very efficient. MDV integration can occur in all chicken chromosome classes, including macro-, intermediate- and micro-chromosomes without a preference for the either the p or q arm [22]. The presence of the virus genome in host telomeres has been confirmed by various techniques including fluorescent in situ hybridization (FISH) and pulsed-field gel electrophoresis (PFGE) [21,23,24,25,26]. Integration into host telomeres has also been shown for other herpesviruses, which will briefly be addressed within this review. In recent years, several advances have been made that have expanded our understanding of the integration mechanism and these will be discussed below.

## 3. MDV Telomeric Repeats

**Figure 1:**
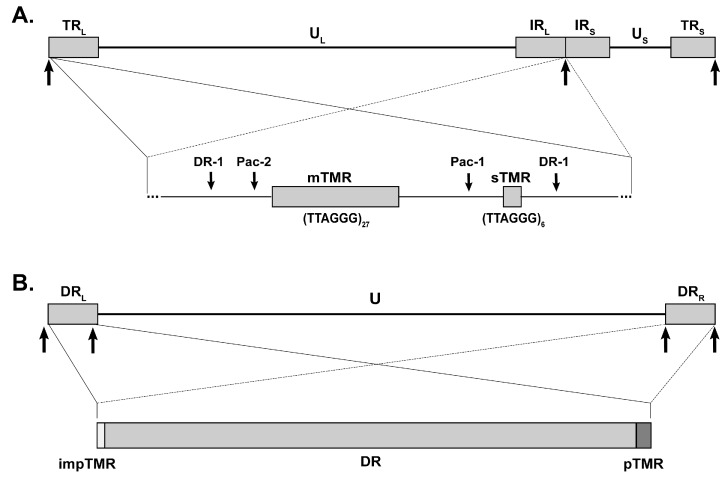
Overview of the Marek’s disease virus (MDV) and human herpesvirus 6 (HHV-6) genome showing the viral telomeric repeats**.** (**A**) Schematic representation of the MDV genome with a focus on the a-like regions containing the multiple telomeric repeats (mTMR) and the short telomeric repeats (sTMR); (**B**) Schematic representation of the HHV-6 genome with a focus on the direct repeat region (DR) containing the imperfect TMR (impTMR) and perfect TMR (pTMR) at the left and right genomic termini, respectively.

MDV has a double-stranded DNA genome of approximately 180 kbp. Its class E genome consists of two unique regions (U_S_ and U_L_) that are flanked by terminal (TR_L_ and TR_S_) and internal (IR_L_ and IR_S_) inverted repeat regions (Figure 1A) [27]. TMR are located within the a-like sequences that are present at both ends of the linear genome as well as in an inverted orientation at the IR_L_-IR_S_ junction [28]. Each a-like sequence harbors two telomeric repeat arrays, multiple telomeric repeats (mTMR), with a variable number of repeats, and short telomeric repeats (sTMR), with a fixed number of 6 repeats (Figure 1A). Both mTMR and sTMR regions are located adjacent to the conserved packaging signals (pac-1 and pac-2) and the genome cleavage site (direct repeat 1, DR-1), both of which are essential for virus replication [29]. This genomic arrangement has been present in all MDV strains sequenced to date. 

We have recently addressed the role of MDV TMR in virus replication, integration and tumor formation [19]. Both mTMR and sTMR were replaced with either structurally similar (TAAGGC)n or completely scrambled repeats (ACGACA)n. Mutation of the TMR did not affect lytic replication in vivo or in vitro; however, it severely impaired viral integration, pathogenesis and tumor formation in infected chickens. Intriguingly, a few animals infected with the mutant viruses still went on to develop disease and tumors. Tumor cells from these animals harbored only a single MDV integration site that was not located in host telomeres, but elsewhere in the chromosome [19]. Furthermore, this particular type of integration did not occur as a single virus genome but as concatemers, suggesting that the virus genome replicated prior to integration. The observation that the virus genome was integrated in all tumor cells even upon mutation of the TMR, led us to conclude that integration is a critical prerequisite for MDV-induced tumor formation as it ensures faithful maintenance of the virus genome and its oncogenes in replicating host cells. Moreover, integration into host telomeres also allowed efficient mobilization of the virus genome during reactivation. In contrast, virus genomes integrated elsewhere in the host genome reactivated poorly [19], highlighting that integration into host telomeres is beneficial for virus reactivation. 

To determine the role of the individual TMR arrays, we deleted only the mTMR sequences. Viruses lacking the mTMR sequences were severely impaired in viral integration, pathogenesis, tumor formation and reactivation [19]. This phenotype was comparable to the mutant viruses harboring the mutated TMR, indicating that the mTMR plays a major role in the MDV genome integration into host telomeres. In addition, it has recently been demonstrated that the sTMR has a dual function in the MDV life cycle [23]. Deletion of sTMR abrogated MDV replication, as these sequences serve as a spacer between the packaging signal pac-1 and the DR-1 cleavage site to ensure proper cleavage and packaging of the viral genome into the capsid. Truncation of the sTMR confirmed that the exact length of the sTMR is crucial for efficient MDV replication [23]. Mutation of the sTMR revealed that the exact sequence is not important for the production of progeny virus. However, mutation of the sTMR reduced integration frequency and impaired MDV pathogenesis and tumor formation [23]. Taken together, this data demonstrates that both mTMR and sTMR play an important role in MDV integration, and that this process is crucial for pathogenesis and tumor formation. 

## 4. Other Telomere Herpesviruses

**Figure 2:**
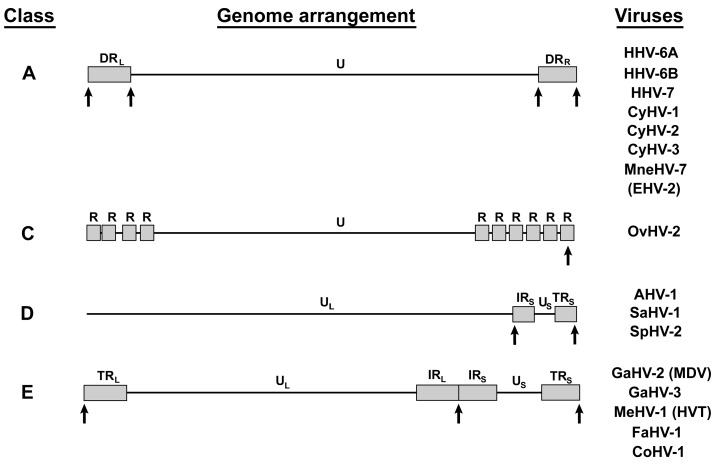
Overview of telomere-containing herpesviruses. Genome classes and structure of indicated herpesviruses are shown based on the NCBI reference sequences. The regions containing TMR are highlighted with arrows. Abbreviations: human herpesvirus 6A (HHV-6A), human herpesvirus 6B (HHV-6B), human herpesvirus 7 (HHV-7), cyprinid herpesvirus 1 (CyHV-1), cyprinid herpesvirus 2 (CyHV-2), cyprinid herpesvirus 3 (CyHV-3) *Macaca nemestrina* herpesvirus 7 (MneHV-7), equine herpesvirus 2 (EHV-2), ovine herpesvirus 2 (OvHV-2), anatid herpesvirus 1 (AHV-1), saimiriine herpesvirus 1 (SaHV-1), spheniscid herpesvirus 2 (SpHV-2), Marek’s disease virus (MDV), gallid herpesvirus 3 (GaHV-3), meleagrid herpesvirus 1 (MeHV-1), falconid herpesvirus 1 (FaHV-1) and columbid herpesvirus 1 (CoHV-1).

Aside from MDV, a number of herpesviruses genomes harbor TMR arrays identical to the host telomere sequences (Figure 2). To date, 17 of the 83 full length herpesvirus genomes available on NCBI (taxid 548681) contain TMR arrays (20.5%). These include members from all *Herpesviridae* subfamilies, the *Alpha-*, *Beta-* and *Gammaherpesvirinae* that infect either humans, other mammals or birds. In addition, three species of the *Alloherpesviridae* that infect various fish species contain TMR arrays. TMR-containing herpesviruses are genetically very distinct, and possess either class A, C, D or E genomes (Figure 2), as reviewed previously [13]. Most TMR-containing herpesviruses have a class A genome (8/17; 47.1%) and harbor TMR arrays at both ends of the direct repeat (DR) region. This ensures the presence of TMR at either end of the linear virus genome (Figure 2). The only exception is equine herpesvirus 2 (EHV-2), which harbors TMR arrays within the DR region. To date, only one TMR-containing herpesvirus with a class C genome has been discovered. This ovine herpesvirus 2 (OvHV-2) harbors a TMR array within the R10 repeat region at the right end of the genome (Figure 2). Three herpesviruses with a class D genome (3/17; 17.6%) contain the TMR arrays at the terminal ends of the inverted repeat short regions (IR_S_ and TR_S_) flanking the unique short region (U_S_). Finally, five class E herpesviruses harbor TMR arrays (5/17; 29.4%) that are present in the a-like sequences at the ends of the virus genome and the IR_L_-IR_S_ junction as described for MDV.

So far, integration into host chromosomes has been shown for six of the telomere herpesviruses, namely: MDV, HHV-6A, HHV-6B, HHV-7, GaHV-3 and MeHV-1 [19,25,30,31] (and unpublished data). The integration sites of these lymphotropic herpesviruses have been detected at the end of chromosomes, the region containing the host telomeres. For MDV, HHV-6A and HHV-6B, integration has been specifically mapped to the host telomeres as reviewed recently [13,32].

HHV-6A and HHV-6B are two closely related betaherpesviruses. HHV-6B infects most humans during early childhood, causing a febrile illness with a rash (*roseola infantum*), while the level of HHV-6A infection and pathogenic burden remain less well understood [33,34,35]. Upon primary infection, both viruses establish latency, allowing them to persist in the host for life. As mentioned above, HHV-6A/B have recently been shown to integrate into the telomeres of latently infected cells [30,36,37,38,39], while no circular episomes could be detected. In addition, the virus can also integrate into germ cells, resulting in vertical transmission, with these individuals harboring the integrated virus in every nucleated cell within their body. This condition is termed inherited chromosomally integrated HHV-6A/B (iciHHV-6) and is present in about 1% of the human population [40,41,42,43,44,45,46,47]. Interestingly, germline integration of MDV has not been observed yet. Whether this apparent discrepancy between MDV and HHV-6 is due to differences in tissue tropism or virus biology remains to be investigated. HHV-6A/B can reactivate from latently infected cells in iciHHV-6 patients, which is associated with several diseases, including encephalitis and graft rejection upon transplantation [48,49]. Similar to MDV, the HHV-6A/B genomes harbor two distinct TMR arrays at either end of the DR regions (Figure 1B and Figure 2). At the right genome end are the perfect TMR (pTMR) that are identical to the human telomere sequence. The left terminus of the genome contain the imperfect TMR (impTMR) consisting of telomeric repeats that are interrupted by related nucleotide hexamers [50,51]. We recently addressed the role of the HHV-6A pTMR and impTMR in virus replication and integration [26]. Deletion of both pTMR and impTMR in the HHV-6A genome revealed that the telomere sequences are dispensable for virus replication. However, integration was severely impaired upon deletion of the TMR, highlighting that the viral telomere sequences also mediate integration for HHV-6A. Deletion of the individual TMR regions revealed that the pTMR plays a major role in integration, while the impTMR only plays a minor role in this process [26].

It has recently been demonstrated that HHV-7 is also able to integrate in vitro and in vivo [31]. Infected SupT1 cell clones harbored the integrated virus genome that colocalized with host telomeres when analyzed by FISH. This quiescent virus genome could be reactivated upon trichostatin A and hydrocortisone treatment resulting in productive virus replication. In addition, telomere-associated HHV-7 was discovered in two patients using this FISH technique. The detection of one viral genome per host cell in peripheral blood cells and hair follicles suggested that this could reflect a germline integration event. However, this work represents the first observation of potential inherited HHV-7 and more research is required to fully understand HHV-7 integration, as well as its potential to enter the germline. 

McPherson and colleagues recently addressed if GaHV-3 and MeHV-1 (aka. herpesvirus of turkeys; HVT) can integrate into host chromosomes [25]. GaHV-3 (SB-1 strain) and HVT (FC-126 stain) also belong to the genus *Mardivirus* and are frequently used as vaccines against MDV [52]. Upon infection of chickens with HVT, the authors could detect the integrated virus genome in infected spleen cells; however, only very few metaphase chromosomes harboring the virus genome were detected. The data was unfortunately only shown in a table while no FISH images were provided to visualize HVT integration [25]. Similarly, possible GaHV-3 integration events were only detected in cells that were either undergoing lytic replication or reactivation at the time points analyzed [25]. Future studies should set out to confirm the integration of GaHV-3 and HVT, determine if it occurs in host telomeres and elucidate if the virus genome is maintained this way in latently infected cells.

## 5. Factors Involved in Telomere Herpesvirus Integration

Several cellular and viral factors have been proposed to be involved in the integration process. Two viral factors that could facilitate MDV integration are UL12 and UL29. They are homologues of the herpes simplex virus 1 (HSV-1) genes UL12 and UL29 (ICP8), which encode a 5′-3′ exonuclease and a single-strand DNA binding protein respectively. Both of these viral proteins have been shown to facilitate recombination during replication of the HSV-1 genome and act through a single strand annealing (SSA) DNA repair pathway [53,54,55]. Whether the functions of these genes are conserved for MDV remains to be elucidated. However, if the functions are conserved, perhaps MDV has co-opted this recombination machinery to integrate its genome into host telomeres.

Another viral protein that could facilitate integration of HHV-6A and HHV-6B is the putative viral recombinase U94. U94 is an orthologue of the adeno-associated virus 2 (AAV-2) integrase (Rep68), and is highly conserved amongst all HHV-6 strains [56]. Expression of U94 could facilitate replication of a mutant adeno-associated virus 2 (AAV-2) lacking Rep68, suggesting that both proteins have similar functions [56,57]. Furthermore, the U94 protein has recently been shown to possess all functions required for homologous recombination [58,59]. Recent work by Wallaschek and colleagues has demonstrated that HHV-6A lacking U94 could efficiently integrate into host telomeres, suggesting other factors can complement the loss of U94 during HHV-6A integration [60]. MDV and other telomere herpesviruses do not encode an orthologue of U94, suggesting that this gene was acquired during later stages in herpesvirus evolution.

Avian and mammalian cells encode two central recombinases involved in homologous DNA recombination (HR), Rad51 and DMC1. While DMC1 is restricted to meiotic cells [61], Rad51 is active in somatic cells and is essential for DNA damage repair (DDR) by HR [62,63,64]. Moreover, cells also contain Rad52, a DDR protein involved in homologous sequence repair by SSA [65]. Due to the cellular functions of these proteins it is possible that they could influence herpesvirus integration, although whether or not they do remains to be determined. An interesting final thought is that telomere-integrating herpesviruses may have remained promiscuous with regards to their route into telomeres, and could potentially utilize the highlighted pathways in a redundant manner.

## 6. MDV Telomerase RNA (vTR)

**Figure 3:**
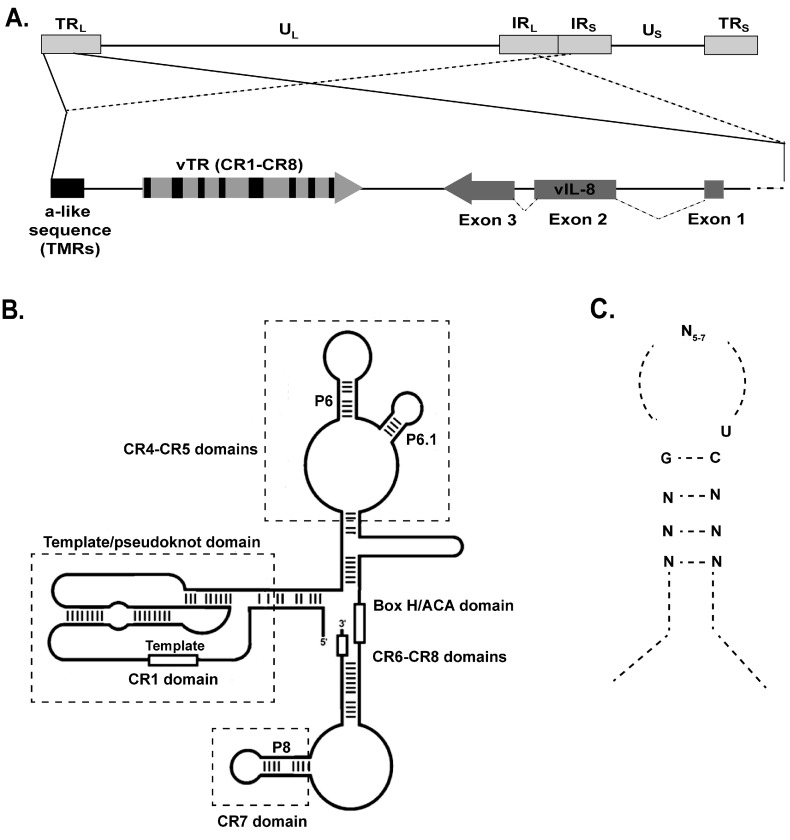
MDV genome overview showing the viral telomerase RNA (vTR) region. (**A**) Schematic representation of the MDV genome with a focus on the regions harboring the TMR array, vTR (443 bp) with its eight conserved regions (CR1-CR8) and the three exons of the neighboring viral interleukin-8 (*vIL-8*) gene; (**B**) Secondary structure of the MDV vTR. The conserved domains of vTR are shown that are also present in all cellular TRs; (**C**) Consensus pattern of the EBER-1 RpL22 binding sites.

The telomerase complex protects telomeres from shortening via the addition of telomeric repeats at the ends of host chromosomes [66]. Telomerase contains two main components; the catalytic subunit telomerase reverse transcriptase (TERT), and the telomerase RNA (TR or TERC), which serves as a template for the addition of telomeric repeats. Telomerase activity is absent in most somatic cells, but is active in stem cells and most human cancers [67]. While TERT is the limiting factor of telomerase activity [68], the role of the constitutive expression of TR in cancer development remains poorly understood. As mentioned above, MDV encodes a TR (vTR) that shares 88% sequence identity with the chicken telomerase RNA (chTR) [14]. The MDV genome harbors two copies of the vTR gene in the TR_L_ and IR_L_ regions that are located between the TMR and the vIL-8 gene (Figure 3A) [14]. vTR interacts with chicken TERT, resulting in higher telomerase activity when compared to chTR. It also restored telomerase activity in murine cells lacking endogenous mouse TR [14]. In general, vTR consists of four main structural domains that are highly conserved in all TRs (Figure 3B) [14]. The pseudoknot (core) domain harbors the template sequence (CR-1) for extension of the telomeric repeats [69], the CR4-CR5 domain is essential for proper assembly with TERT [69] and the H/ACA box and CR-7 domain are responsible for TR stability and localization [14,70,71]. Overexpression of vTR has been shown to enhance transformation of DF-1 chicken cells [15], indicating that vTR could play a role in MDV-induced transformation.

## 7. Role of vTR in Tumorigenesis

To investigate if vTR has a role in MDV-induced malignant transformation, Trapp and colleagues generated MDV mutants that lack most of the vTR gene (∆CR1-CR4) [15]. These mutant viruses replicated in a manner comparable to wild type virus in vitro and in vivo in the absence of vTR, underlining that vTR is dispensable for lytic replication [15]. Importantly, disease development and tumor formation in the infected chickens was severely impaired in the absence of vTR [15]. Furthermore, deletion of vTR resulted in reduced tumor sizes and dissemination. A comparable phenotype was observed upon deletion of the essential CR1-2 domain. Overexpression of vTR not only increased cell proliferation but also upregulated the levels of cell-surface adhesion molecules such as integrin α-V [15], suggesting that vTR could have functions beyond its role in telomerase activity. 

## 8. Telomerase Independent Functions of vTR

To investigate if the tumor promoting functions of vTR are dependent on the telomerase activity, we generated a mutant MDV where the base paring of the P6.1 stem of vTR was disrupted [16]. Mutation of the P6.1 stem interfered with proper TR-TERT interaction and abrogated telomerase activity [16]. MDV P6.1 mutant viruses replicated with similar kinetics to wild type virus in vitro and in vivo [16]. Intriguingly, only the onset of disease was delayed, while lymphoma formation was not altered by this mutation [16]. Furthermore, tumor dissemination was also comparable to the wild type [16], suggesting that the tumor promoting functions of vTR are independent of its role in the telomerase complex. We also demonstrated that vTR can interact with the cellular protein RpL22 [72], which plays an important role in T-cell development and lymphoma formation [73,74]. RpL22 not only interacts with vTR, but also the Epstein-Barr virus-encoded RNA 1 (EBER-1) [72]. EBER-1 has been shown to contain three RpL22- binding sites [72]. Evaluation of the RpL22 binding sites revealed a consensus pattern consisting of a stem loop structure with a G-C at the stem neck followed by a U (Figure 3C) [75]. vTR also harbors two stem-loops (P6 and P8) that contain this consensus pattern [76,77], but whether these stem loops are indeed RpL22-binding sites remains to be determined. Overexpression of both vTR and EBER-1 result in a re-localization of RpL22 [16], however the biological consequences of this process are still unknown. Intriguingly, overexpression of human TR (hTR) has recently been shown to possess anti-apoptotic functions in human immune cells. Moreover, this anti-apoptotic effect of hTR was independent of the telomerase complex [78]; although it remains unknown if vTR also exhibits this activity.

## 9. vTR Expression Levels

vTR is highly expressed during all stages of the virus lifecycle including lytic virus replication, persistent infection and in MDV-induced tumor cells [15]. It is the most abundant viral transcript detected in MDV-induced tumor cells and has higher expression than chTR in infected cells, which is likely due to differences in their promoters [70,79,80]. The vTR promoter has additional AP-1, E-boxes, and EBS transcription factor binding sites, which are not present in the chTR promoter [81]. To determine if the vTR promoter and the corresponding expression levels are crucial for the oncogenic properties, Chbab and colleagues engineered a MDV mutant in which the vTR promoter was replaced by the promoter of chTR [80]. Expression levels of vTR were 3-fold lower under control of the chTR promoter compared to the native promoter. Interestingly, this promoter exchange did not affect virus replication, but severely impaired disease development [80]. In addition, tumor incidence in the infected chickens was significantly reduced when compared to wild type virus [80]. Replacement of the vTR promoter also resulted in smaller tumors and less tumor dissemination, demonstrating that the high vTR expression levels are crucial for efficient tumor formation [80].

## 10. vTR-Based Vaccines

Previous studies have revealed that mutation of the hTR template sequence led to the incorporation of incorrect telomeric repeats into telomeres, resulting in telomere instability and ultimately apoptosis [82]. Moreover, expression of vTR with the template sequence mutated from AATCCCAATC to ATATATATAT (AU5) efficiently reduced cancer cell proliferation [69]. To investigate the effects of mutation of the vTR template on disease and tumor formation, we generated an MDV mutant harboring the AU5 mutation (vAU5) [69]. Disease and lymphoma formation was completely abrogated in chickens infected with the vAU5 mutants. To confirm that the absence of the tumors was specifically due to incorporation of mutant telomeric repeat sequences into host telomeres by the telomerase, the P6.1 stem-loop was mutated to abrogate the incorporation of the AU5 mutant vTR into the telomerase complex [16]. Abrogation of the vTR-TERT interaction restored tumor formation, confirming that the observed effect of the AU5 mutation is dependent on its incorporation into the telomere complex, resulting in introduction of incorrect telomeric repeats into host telomeres and apoptosis. Furthermore, vaccination-challenge experiments revealed that the vTR-AU5 mutants efficiently protected resistant and susceptible chicken lines from a fatal challenge with a very virulent MDV strain [69]. The conferred protection was at least as good as the MDV gold standard vaccine CVI988/Rispens; however, large scale vaccination trials are needed to address if the AU5 mutant virus provides enhanced protection compared to the current vaccines.

## 11. Conclusions

A number of viruses can integrate their genome into host chromosomes during their virus lifecycle.

Some viruses such as adeno-associated viruses (AAV) integrate into human genome at a specific site (AAVS1), while others such as retroviruses can integrate across the entire host genome [83]. MDV and several other herpesviruses have been shown to specifically integrate their genome into the host telomeres of latently infected cells. This facilitates replication of the integrated virus genome with the chromosomes in dividing host cells, which in turn ensures maintenance of the virus genome in the host for life. TMR sequences in these herpesvirus genomes are required for telomere integration, although the exact mechanism and factors required for integration remain to be fully defined. The host telomere sequences appear to provide the optimal target for herpesviruses as: (i) long homologue sequences are present in every chromosome to enhance integration efficiency; (ii) telomeres repress expression of adjacent genes [84], and could therefore aid in silencing the virus genome during the establishment of latency; and (iii) the natural shortening of the telomeres could trigger virus reactivation. This could be either by relieving suppression of viral gene transcription or inducing telomere-telomere recombination, resulting in release of the virus genome from the integration site. In the case of MDV, integration plays a crucial role in MDV-induced tumor formation, underlining the necessity of maintaining the virus genome including its oncogenes to drive cellular transformation.

In addition to the TMR, the MDV genome also encodes vTR that has a high sequence identity to the host chTR, suggesting that the gene was acquired from the host genome during evolution. vTR is expressed during lytic and latent stages of MDV infection and is the most abundant transcript in MDV-transformed cells. Furthermore, it has been shown that overexpression of vTR plays an important role in tumor formation. Intriguingly, overexpression of human telomerase RNA (hTR) has been shown to possess anti-apoptotic functions that are independent of its role in telomerase activity. Similarly, telomerase-independent functions of vTR are crucial for efficient tumor formation; however, the exact mechanism behind this remains unknown. The interaction between vTR and RpL22 could play an important role in tumor formation and should be addressed in future studies. Finally, further work remains to be done in order to understand the contribution of vTR and cellular TRs towards the development of cancer.
